# State-of-the-Art Review: Technical and Imaging Considerations in Novel Transapical and Port-Access Mitral Valve Chordal Repair for Degenerative Mitral Regurgitation

**DOI:** 10.3389/fcvm.2022.850700

**Published:** 2022-04-12

**Authors:** Romy M. J. J. Hegeman, Livia L. Gheorghe, Thomas L. de Kroon, Bart P. van Putte, Martin J. Swaans, Patrick Klein

**Affiliations:** ^1^Department of Cardiothoracic Surgery, St. Antonius Hospital, Nieuwegein, Netherlands; ^2^Hospital Universitario Puerta del Mar, Cádiz, Spain; ^3^Department of Cardiology, St. Antonius Hospital, Nieuwegein, Netherlands

**Keywords:** minimally invasive mitral valve surgery, minimally invasive MV repair, mitral valve surgery, mitral valve repair, Harpoon, Beating Heart Mitral Valve Repair, transapical, chord implantation

## Abstract

Degenerative mitral regurgitation (DMR) based on posterior leaflet prolapse is the most frequent type of organic mitral valve disease and has proven to be durably repairable in most cases by chordal repair techniques either by conventional median sternotomy or by less invasive approaches both utilizing extracorporeal circulation and cardioplegic myocardial arrest. Recently, several novel transapical chordal repair techniques specifically targeting the posterior leaflet have been developed as a far less invasive and beating heart (off-pump) alternative to port-access mitral repair. In order to perform a safe and effective minimally invasive mitral chordal repair, thorough knowledge of the anatomy of the mitral valve apparatus and adequate use of multimodality imaging both pre- and intraoperatively are fundamental. In addition, comprehensive understanding of the available novel devices, their delivery systems and the individual procedural steps are required.

## Introduction

Degenerative mitral regurgitation (DMR) is the second-most frequent valvular heart disease in Europe and the United States, affecting approximately 10% of the general population (1, 2). DMR is the predominant cause of primary mitral regurgitation (MR) (3). In accordance with current guidelines, mitral valve (MV) surgery is recommended in case of symptomatic severe chronic primary MR when the surgical risk is acceptable (2). When surgery is considered, MV repair is the intervention of first choice (2). In recent decades, several techniques for minimally invasive mitral valve surgery (MIMVS) have been developed and have conquered territory in the field of mitral surgery (4). Multiple studies have reported equally durable repair with comparable safety after MIMVS compared to conventional MV repair through median sternotomy. Benefits of MIMVS by port-access approaches are a shorter length of hospital stay, shorter postoperative ventilation times and shorter intensive care unit stay (5). From an even less invasive perspective, transcatheter MV repair can be considered as a safe alternative in patients with contraindications for surgery or high operative risk, with edge-to-edge repair being the most applied and investigated technique (2). However, since posterior leaflet prolapse, especially of the P2 scallop, is found in approximately 75% of patients with severe degenerative MR (6) and is also highly repairable, interest in transapical treatment options that solely target P2 has rapidly increased in the past decade. The HARPOON TDS-5 (Edwards Lifesciences, Irvine, CA) and the Neochord DS 1000 (NeoChord, Inc., St. Louis Park, MN) are the current available transapical chordal implantation systems that have gained Conformitié Européenne (CE) mark approval.

In this review we focus on both the transapical approach of mitral valve repair using a novel chordal implantation HARPOON device, as well as on minimally invasive port-access mitral chordal repair. We will describe important anatomical considerations that are relevant for the procedure and offer a practical guide to periprocedural imaging.

## Preoperative Echocardiographic Imaging Considerations for the Evaluation of Degenerative Mitral Regurgitation

When a patient is opted for surgical correction of the mitral valve (either via conventional sternotomy, through a minimally invasive surgical approach or a transapical or transcatheter approach) a detailed evaluation of the MV must be available.

The mitral annulus (MA) can be measured in parasternal long-axis (PLAX), apical four-chamber (AP4CH), apical two-chamber (AP2CH), and apical three-chamber (AP3CH) views. The measuring points should be located at the transition between the MV leaflets and the left atrium (LA). When the annulus is dilated, the anteroposterior (AP) diameter will predominantly increase, as represented in the PLAX, AP2CH, and AP3CH views. Special attention should be paid to mitral annular calcification (MAC), which—if present—can be seen in all MV views. However, MAC is especially well seen in the PSAX view (7).

When the posterior leaflet base is displaced superiorly, mitral annular disjunction (MAD) could be present. MAD, characterized by a wide separation between the atrium-MV junction and the left ventricular (LV) attachment, can be detected both on TTE and TEE (8).

The mitral leaflets can be adequately visualized in the PLAX and parasternal short-axis (PSAX) view and in the apical views. The PSAX view visualizes the surface of both leaflets and the closure-line can easily be followed from the anterolateral to the posteromedial commissure. The PLAX view cuts through the middle of the MV, thus picturing A2 and P2. In the AP4CH view the valve is transected in a more mediolateral manner, therefore visualizing A3 and P1. The A2 and P2 scallop are both visible In AP5CH, whereas AP2CH shows A1, P2, and P3. The PSAX view at the level of the MV is well suited for identification of clefts, which is especially relevant when the patient is scheduled for chord implantation (7).

Prolapse of the MV is best identified in the AP3CH and PLAX views, because these transect the AP plane. An eccentric MR-jet is seen in case of significant prolapse, directed away from the prolapsing leaflet. Although 2D-images also enable identification of flail, it is hard distinguish the exact origin (e.g., chordal rupture or chordal elongation) on 2D-images. Therefore, supplementary TEE is valuable. In case of chordal rupture, an eccentric jet is classically seen from the flail leaflet toward the wall of the LA (7). Relevant mitral prolapse measurements include the billow volume, billow height, length from the anterolateral papillary muscle to coaptation and length from the posteromedial papillary muscle to coaptation (8).

If a patient is accepted for chordal repair, it is useful to determine the leaflet-to-annulus index (LAI), defined as the ratio between the sum of anterior leaflet length and posterior leaflet length over anteroposterior length (9). As the LAI represents the amount of excessive tissue, it is also considered as an expression of potential leaflet-annulus mismatch (10).

When examining the severity of MR, several diagnostic echocardiographic parameters can be useful. First of all, the vena contracta should be determined. The vena contracta can be measured in the PLAX and AP3CH view, or in transversal planes on TEE. A vena contracta-width of ≥ 7 mm is suggestive for severe MR. Additionally, the jet-surface needs to be evaluated. A jet-surface of > 10 cm^2^ is representative for severe MR (or > 8 cm^2^ in TEE), as well as a jet-surface/LA-surface ratio of > 40%. Furthermore, Doppler evaluation of the pulmonary vein flow can be assessed in AP4CH or on TOE. When systolic backflow into one of the pulmonary veins is present, severe MR is likely (90% chance). Moreover, evaluation of the flow convergence zone on both TTE and TEE can attribute to the diagnosis of severe MR (in case of a radius > 0.9 cm). Other helpful complementary determinants in the diagnosis and evaluation of severe MR are increased mitral inflow velocity (>1.2 m/s) and enlargement of the LA and LV. Quantitative parameters that can be used for the evaluation of the severity of MR can be determined with the Proximal Isovelocity Surface Area (PISA) method (e.g., regurgitant volume and effective regurgitant orifice) (7).

## Minimally Invasive Mitral Valve Repair

### Anatomical Considerations

Anatomic MV landmarks that should be kept in mind include the left and right trigones (at the fibrous border between the mitral and aortic valves), the anterolateral and posteromedial commissures, the leaflet tips (corresponding to the line of each leaflet free edge), the posterior annular midpoint and the papillary muscle tips (11). Due to the anatomical location of the left atrium and the mitral valve, a direct left atrial approach via right-anterolateral thoracotomy provides adequate exposure for surgical repair. Important considerations for repair are elevated right hemi-diaphragm and the presence of obesity, as this could impact the exposure. During mitral valve surgery, extra caution is required at the anterolateral commissure (e.g., in ring annuloplasty) because of the circumflex artery, as the distance between these structures at this point can be as little as 1 mm (12). Additionally, severe annular calcifications can form a challenge as extensive decalcification and reconstruction of the mitral annulus is more difficult through a minimally invasive approach (13).

### Imaging-Derived Patient Selection and Patient Preparation

Minimally invasive MV repair (MIMVr) via a right anterolateral thoracotomy can be performed in the majority of patients with an indication for MV repair (13), as determined with echocardiography (e.g., with Philips EPIQ). Although MIMVr is not solely reserved for the repair of posterior leaflet prolapse, patients with isolated P2 prolapse are particularly good candidates for MIMVr (13). Patients with or without concomitant tricuspid disease, atrial septal defect, and atrial fibrillation can be considered for a port access approach (13, 14). However, a conventional median sternotomy is often preferred in patients who require extensive concomitant procedures such as coronary bypass graft surgery, aortic valve replacement or ascending aorta replacement (13). Contra-indications for MIMVr are summarized in Table 1.

**TABLE 1:** Contra-indications for MIMVr.

Contra-indication
– Previous right thoracotomy
– Severe mitral annular calcification
– Moderate or severe aortic valve regurgitation
– Extensive concomitant surgery requiring a median sternotomy (e.g., CABG, AVR, ascending aorta replacement)
– Kinking of the femoroiliac vessels
– Aortic root/ascending aorta calcification and/or dilatation
– Pleural adhesions
– Severe peripheral vascular disease
– Severe irreversible pulmonary hypertension (>60 mm Hg)
– Severe pulmonary disease
– Reduced RVEF/right ventricular dysfunction
– Recent stroke or TIA
– Severe liver dysfunction
– Significant coagulopathy
– Mitral valve endocarditis

*AVR, aortic valve replacement; CABG, coronary artery bypass grafting; CT, computed tomography; RVEF, right ventricular ejection fraction; TEE, transesophageal echocardiography; TIA, transient ischemic attack.*

The use of a combination of both computed tomography (CT) scanning of the total aorta and detailed echocardiography can add value to the pre-operative work-up.

Although TTE is used to complete diagnosis in most patients, TEE is strongly recommended as it gives a clear image of the mechanisms contributing to MV disease and the specific site that causes MR. The presence of aortic regurgitation should be assessed on echocardiography preoperatively, as it can cause difficulties with cardioplegia administration (13). Furthermore, patients with MV endocarditis can be treated with MIMV surgery, but the presence of peri-annular abcesses should be evaluated echocardiographically or with CT-scanning preoperatively as it forms a contra-indication for this approach (13).

As mentioned above, a detailed pre-operative analysis based on CT-scanning can add value both in determination of patient-eligibility and procedural planning of MIMVr. With visualization of the femoroiliac vessels on CT, the suitability for peripheral cannulation can be assessed. Kinking of these vessels and/or severe peripheral atherosclerosis or calcifications can specifically cause challenges for femoral cannulation. Moreover, arteries with a luminal diameter < 7 mm are poor candidates for cannulation. Therefore, minimal luminal diameter, atherosclerotic burden and tortuosity or abnormal angulation of femoroiliac vessels can be determined on CT (14). By visualizing the central veins on CT, venous stenoses and occlusions, anomalies or clots can be identified that could impair venous drainage during cardiopulmonary bypass (CPB) (14). As retrograde CPB perfusion via the femoral artery may be problematic and dangerous in the presence of descending aortic aneurysm, aortic dissection, or aortic thrombus, it can add to patient-safety to check this preoperatively on CT. Furthermore, extensive calcifications of the ascending aorta or ascending aorta dilation of more than 4.5 cm can complicate aortic clamping and administration of antegrade cardioplegia (13).

In the pre-operative work-up, the presence of severe mitral annular calcification should be evaluated (either on echocardiography or CT) as it is accompanied by high procedural risk, and extensive decalcification and reconstruction of the mitral annulus is very challenging through a minimally invasive approach (13).

The position of the right hemidiaphragm can be analyzed both on CT (14) and chest X-ray to determine the appropriate site for port placement.

Additionally, CT-scanning can provide geometric and anatomical data on the mitral valve and subvalvular apparatus that can be taken into consideration in preprocedural planning. Complementary three-dimensional (3D) volume rendered images obtained from the preoperative CT scans may allow the surgeon to analyze the patient’s anatomy and determine optimal locations of port access, but are not often used in clinical practice for this purpose (14).

### Procedural Technique

In our high-volume MIMVr center, our main strategy to repair isolated prolapse of the pMVL is by the “respect” technique described by Patrick Perrier (15) in which the prolapsing pMVL segment is corrected by implantation of polytetrafluoroethylene (PTFE) neochordae from the fibrous parts of the papillary muscle complexes in combination with an annuloplasty ring. We will therefore focus on this technique and we will describe our procedural technique step-by-step, based on the Leipzig approach (16, 17) and also the (premeasured) loop technique as described by von Oppell and Mohr (18).

#### Patient Set-Up

**FIGURE 1:**
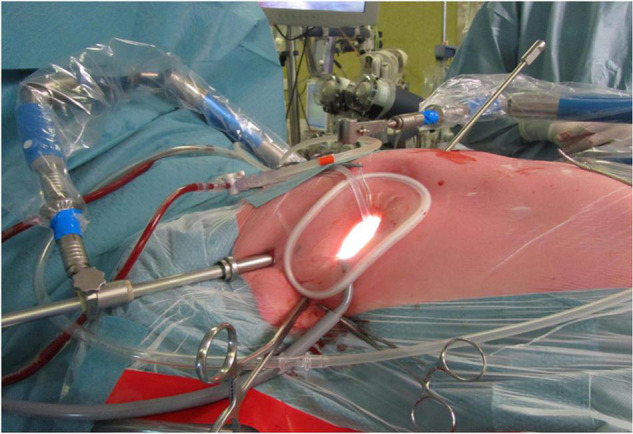
The set-up for a minimally invasive port-access approach through a right anterolateral thoracotomy, as performed in the Sint Antonius Hospital Nieuwegein. The patient is positioned in a 30* left lateral position with the right arm placed posteriorly, with a sand bag under de right scapula. A soft tissue retractor is used without additional rib spreading. Depicted are the atrial roof retractor, camera port and aortic cross-clamp (from medial to right lateral).

The procedure is performed under general anesthesia with a double-lumen endotracheal tube. External defibrillator pads and meticulous control of activated clotting time (ACT) add to patient safety. The patient should be positioned in a 30* left lateral position with the right arm placed posteriorly (Figure 1). The right hemithorax is elevated by placing a sandbag or inflatable bag under the right scapula. Conventional cross-clamping with a Chitwood clamp only requires a single radial arterial catheter. The use of endoaortic balloon cross-clamping requires surveillance with bilateral radial arterial catheters in order to detect cephalic displacement (19). However, the latter is not often applied in our center and therefore we will not focus on this technique for aortic occlusion.

#### Incision

A 4 cm right anterolateral mini-thoracotomy is made at the fourth intercostal space (ICS) to obtain surgical access. The location of the incision should be just inferolateral to the nipple in men and in the submammary crease in women. A more direct view of the MV can be achieved with a more lateral incision, but substantially increases the distance toward the MV (19). A closer access can be provided with a more medial approach, which could be convenient in more obese patients. If extreme elevation of the right hemi-diaphragm is seen on pre-operative chest X-ray or CT, the targeted location of the incision can be adapted. A soft tissue retractor is used to avoid trauma due to rib spreading (Alexis Wound-protector, Applied Medical, Santa Margarita, CA, United States).

#### Cannulation

Peripheral cannulation is achieved through the femoral artery and vein and should be performed according to the Seldinger’s puncture technique. A 2–3 cm oblique incision is made in the groin. A purse-string suture with 5–0 Prolene is placed at the femoral artery and vein. The femoral vein is cannulated first and a 22 Fr long single venous cannula is inserted (in case of isolated MV surgery). The tip of the venous cannula must lie in the superior vena cava, or IVC with an additional cannula through the RIJV, especially for additional TV interventions. The location of the tip of the venous cannula should be confirmed on TEE. Hereafter, the femoral artery is cannulated with a 14–18 Fr arterial cannula (depending on patient size and arterial diameter) (19). The arterial cannula is fixated to the skin to avoid dislocation. Alternatively, percutaneous peripheral cannulation (with or without the use of sonography) can be performed with the additional use of vessel closure devices.

#### Video-Assisted Endoscopic Monitoring

A video-camera is placed through a 10 mm port in the 2nd or 3rd right ICS (at the right anterior axillary line) directly after accessing the thoracic cavity. A monitor that displays the procedural scopic images should be placed directly in front of the surgeon on the left side of the patient. Carbon dioxide should be delivered through the camera port into the surgical field.

#### Intrathoracic Exposition

Pericardial stay sutures are placed and are brought out by an Endo-Close trocar site closure device (Medtronic) through a stab incision in the right sixth or seventh ICS. The pericardium should be opened 3–4 cm anterior and parallel to the phrenic nerve from the distal ascending aorta toward the diaphragm (19). Consequently, the pericardial stay-sutures can be placed.

#### Aortic Clamping

A Chitwood aortic cross-clamp is placed across the ascending aorta through a 5 mm port in the 3rd right ICS to accomplish aortic occlusion. Alternatively, endo-clamping can be performed. By using a femoral arterial cannula with a side arm, a guidewire and hereafter multi-lumen catheter can be advanced into the ascending aorta under TEE guidance (19, 20). An inflatable balloon is used to accomplish endo-clamping, while antegrade cardioplegia delivery and aortic root venting can simultaneously be achieved (19).

#### Cardioplegia

A purse-string suture is placed for the cardioplegia needle vent with Prolene 4-0 RB. Antegrade crystalloid cardioplegia (approximately 2 L) is delivered directly into the aortic root through the cardioplegia needle. This should be repeated after 90–120 min if necessary. Body temperature should be around 34°C and vacuum-assisted venous drainage should be used throughout the procedure (16).

#### Mitral Valve-Exposition and Repair Technique

A left atriotomy is performed with a paraseptal incision in the Sondergaard groove to access the MV and a left atrial roof retractor is placed to further expose the MV. The prolapse is corrected by implantation of expanded polytetrafluoroethylene (ePTFE) chords fixed to the fibrous tips of the appropriate papillary muscle complexes by a figure of eight (care should be taken not to transfer the midline). Following this, both strands of the chords are fixed to the prolapsing segment of the leaflet and the length is adjusted to correct the prolapse (essentially comparable to the non-diseases primary chords). This is repeated depending on the extent of the prolapsing segment, ensuring each 5 mm of prolapsing segment is secured by a chord on each lateral aspect. A water-test is used to fine-tune the length of the chords after which they are finally tied.

An alternative approach is the implantation of ePTFE chords based on the “Loop technique” of von Oppell and Mohr (18). The required ePTFE length is based on the distance between the posterior leaflet edge and planned site of implantation on the papillary muscle first. The distance can be measured with the use of a ruler or other measuring device, with the normal valve segment either adjacent or on the opposite leaflet to the prolapsing segment as a reference point (18). With the use of a vernier caliper or measuring device as a template, Gore-Tex ePTFE loops can be lengthened according to the premeasured length, by tying a knot over a small pledget. Hereafter the ePTFE suture needles can be passed back through the pledget. The needles can then be passed from anterior to posterior on the respective papillary muscle and tied over a second pledget. The end of the loop is fixated to the atrial side of the prolapsing leaflet segment, by using a second Gore-Tex ePTFE suture and knot the latter on the ventricular surface of the leaflet. The past steps should be repeated for each ePTFE pair (normally 1–3 pairs are used).

Implantation of a semi-rigid annuloplasty ring as described by Carpentier (21), complements the repair. The ring should be sized according to the height of the aMVL. U-stitches (Ticron 2-0) are placed through the annulus one mm outside the junction between the leaflet and the atrium and then through the sewing ring, starting at the anterolateral commissure.

A water-test is conducted to immediately check the MV function after the repair is completed.

#### De-Airing and Decannulation

The vent that is located in the right superior pulmonary vein should now be moved across the MV in order to divide the venting holes in both the LV and the LA. Subsequently, the LA is de-aired by filling it with saline during closure. When the absence of air is confirmed by TEE, the cardioplegia needle vent is removed during weaning of extracorporeal circulation (ECC).

The LA is closed in two layers with a Prolene 3-0 suture. Temporary atrial pacemaker wires are placed.

#### Final Transesophageal Echocardiography Assessment

After completion of the repair, a TEE should be made intraoperatively to assess both the MV and left ventricular function (LVF). When assessing the MV function, the presence of residual MR, coaptation length, inflow-gradient and presence of systolic anterior motion (SAM) should be evaluated. In case of residual MR more than grade 1 (mild), and/or mean inflow-gradient > 5 mmHg and/or systolic anterior motion, a second pump run should be considered (17). Furthermore, the LVF and presence of new regional wall motion abnormalities need to be examined, mostly because of the risk of left circumflex artery occlusion. If the result is satisfactory, CPB-weaning can be initiated.

#### Closure

The pericardium is either approximated with a single suture or left open. Routine closure in layers is performed with a single small caliber left pleural drain (21 Fr).

### Results of Previous Studies

Several different strategies have been reported for the repair of prolapsing pMVL: more classic resection techniques that are based on the technique of Carpentier include triangular or quadrangular resection or plication, annular plication, leaflet sliding, and more recent implantation of ePTFE chords on the prolapsing segments. However, these techniques are always applied in combination with a (semi)rigid annuloplasty ring (17, 22, 23). In our high-volume center, minimally invasive triangular or quadrangular resection and sliding plasty of the annulus were performed in early patients operated before 2009, but from 2009 on isolated prolapsing leaflets are repaired through port-access with the implantation of neochordae combined with an annuloplasty ring (17).

#### Chordal Replacement vs. Leaflet Resection

A systematic review published in 2017 that compared chordal implantation with leaflet resection for isolated pMVL prolapse (predominantly P2) revealed that chord replacement was associated with a lower risk of reoperation (1.1% vs. 4.3%, *p* = 0.0007), equal survival and recurrence of moderate MR, when compared with resection (24). Similar results were reported in a systematic review that was published in 2018 (25).

Additionally, a small difference in transmitral gradient (2.5 vs. 2.8 mmHg, *p* = 0.0004) and orifice area (3.2 vs. 3.0 cm2, *P* = 0.002) was observed in favor of chordal replacement (24). The latter was also confirmed by a recent study of Sakaguchi et al. (26), who showed a lower mean MV gradient of 2.6 ± 1.1 vs. 3.0 ± 1.4 mmHg (*p* = 0.03) and a larger effective orifice area of 1.86 ± 0.48 vs. 1.66 ± 0.47 cm2 (*p* < 0.001) in favor of chordal replacement. Importantly, Sakaguchi also demonstrated that the mean MR grade before discharge was significantly lower in the chordal replacement group than in the resection group (0.22 ± 0.41 vs. 0.35 ± 0.49, *p* = 0.018), based on a combined amount of 291 patients who underwent elective MV repair for isolated pMVL prolapse between 2012 and 2020 (26).

#### Durability of Repair

Previous studies have shown that chordal replacement with ePTFE sutures is a safe, effective and durable technique to correct mitral valve prolapse through conventional median sternotomy, even without tissue resection (27).

Valvular reoperation rates of 0.8% (0.2–1.4%) at 1 year, 2.9% (1.6–4.2%) at 10 years and 4.2% (2.4–6.0%) at 20 years follow-up were reported by David et al. after correction of mitral prolapse with chordal replacement with or without leaflet resection in 746 patients (28). Another large series of 608 patients who underwent MV repair with artificial neochordal implantation revealed 92% freedom from MV reoperation at 15 years (29). Current guidelines state a maximum 1% per patient-year reoperation rate for expert centers (2).

Event-free survival ranged from 97.7% (96.4–98.6%) at 1-year follow-up to 86.3% (83.4–88.8%) at 10 years and 62.9% (56.3–68.5%) at 20 years follow-up (28). Salvador et al. reported a perioperative mortality < 1% and cumulative survival of 84% (75–90%) at 15 years (29).

#### Predictors of Success

A recent study by Bonaros et al. revealed that based on a series of 686 patients with any mitral valve prolapse, the use of ePTFE chords to repair the prolapsing segment was an independent predictor of procedural success in MIMVr (23). Success was defined by successful primary mitral repair without conversion to replacement or to larger thoracic incisions together with the absence of residual MR > mild at discharge and reoperation within 30 days (23). Older age, hypertension and LVEF < 40% has been shown to be predictive of recurrent moderate or severe MR (30). Furthermore, posterior leaflet pathology has been shown to be associated with a higher reoperation-free survival as compared to other localizations (23). In a study conducted by David et al., isolated anterior leaflet prolapse was similarly associated with increased hazard of MV reoperation (28). This might be explained by the more challenging determination of the adequate length of artificial chords in patients with anterior leaflet prolapse (27), emphasizing its use in posterior leaflet prolapse and the use of preformed, fixed-length chordae loops (9).

#### Outcome of Minimally Invasive MV Repair Using Artificial Chordae

Although the use of chordal implantation techniques widely increased over time in the setting of minimally invasive mitral repair, many studies do not isolate the experience with chordae in their reports as it is often part of a broad repertoire of MIMVr techniques (31).

Out of all reports that have described the outcome of chord replacement for MIMVr, the group of Seeburger et al. has described the largest experience in three different reports (32–34). In 2008, they reported the outcome of MIMVr in 1339 consecutive of whom 511 underwent chord replacement with ring annuloplasty (32). Preoperative prolapse of the pMVL was present in 61.5% of study patients. A 30-day mortality of 2.4% was found, together with a survival of 82.6% at 5 years. Freedom from reoperation was 96.3% at 5-year follow-up (32). The same group published a report 1 year later (34), describing the outcome of the loop technique vs. leaflet resection for isolated pMVL prolapse in 670 patients. Freedom from reoperation at 5 years was 98.7%. A larger mitral orifice area and lower mean pressure gradient were found after loop implantation. Additionally, Seeburger and colleagues compared the difference in outcome of MIMVr (with leaflet resection and/or implantation of neochordae, combined with ring annuloplasty) between pMVL and aMVL prolapse (33). Excellent valve function was achieved in the majority of patients, regardless of the repair technique. No significant differences were found between groups regarding long-term survival or freedom from reoperation (33).

In a study conducted by Kuntze et al., 632 patients underwent MVr with Gore-Tex loops for MV prolapse, of whom 522 patients underwent a mini-thoracotomy (35). Loops were used to correct isolated pMVL prolapse in 308 patients. Pooled data showed that MV repair with neochordae resulted in excellent early- and mid-term outcomes for all types of leaflet prolapse. More specifically, survival was 98.6% at 30 days, 97.1% at 1-year in the entire group. Freedom from reoperation was 97.4 ± 1.4% 3 years postoperatively.

Although the studies mentioned above are heterogeneous in composition of both the location of pathology and applied repair technique, it is safe to conclude that in the hands of an experienced MIMVr surgeon, artificial chordal implantation achieves a similar safety and efficacy outcome compared to conventional approach for mitral repair, while reducing surgical trauma (31).

## Transapical Beating Heart Chordal Implantation Systems

Beating Heart Mitral Valve Repair (BHMVR) is based on transesophageal echo-guided transapical implantation of artificial ePTFE chords on the beating heart for patients with severe degenerative mitral valve regurgitation (DMR) based on posterior mitral valve prolapse of the P2 scallop.

This procedure allows minimally invasive surgical anatomical MV repair in patients with suitable anatomy. This technique limits risk and surgical trauma to a minimum, which shortens hospital stay and recovery after surgery. The Harpoon TSD-5 (Edwards Lifesciences, Irvine, CA) and the Neochord DS 1000 (NeoChord, Inc., St. Louis Park, MN) are the current available transapical chordal implantation systems that have gained Conformitié Européenne (CE) mark approval.

## Harpoon Beating Heart Mitral Valve Repair

### Anatomical Considerations

Anatomic LV landmarks include the LV apex, left anterior descending (LAD) coronary artery and the diagonal branches. Due to the anatomical long axis of the (apex of the) LV and the location of the mitral valve, a direct left-ventricular approach via a small left antero-lateral thoracotomy provides sufficient exposure for transapical MV repair. Important considerations are extreme obesity and severe chest wall deformities, as these factors will impact exposure.

### Echo-Derived Patient Selection

**FIGURE 2:**
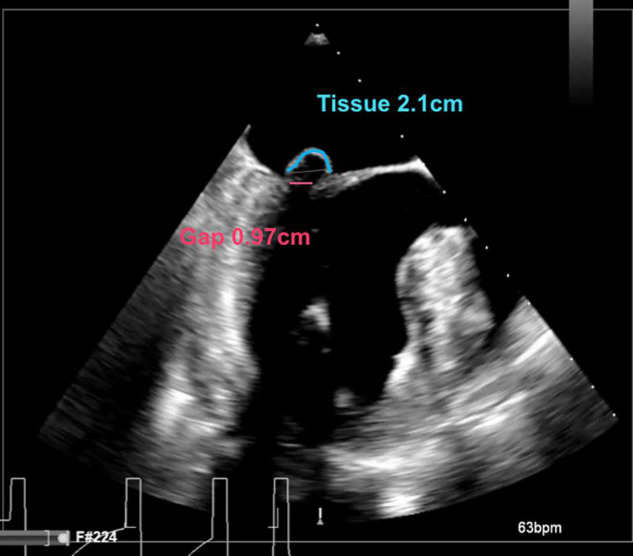
Intraoperative long-axis TEE view showing part of the left atrium, the left ventricle and the left ventricular outflow tract, before repair with the HARPOON device. The measurement of the tissue/gap-ratio is demonstrated. Tissue-length (red), 2.1 cm; gap-length (blue), 0.97 cm. Tissue/gap-ratio = 2.1/0.97 = 2.2.

**FIGURE 3:**
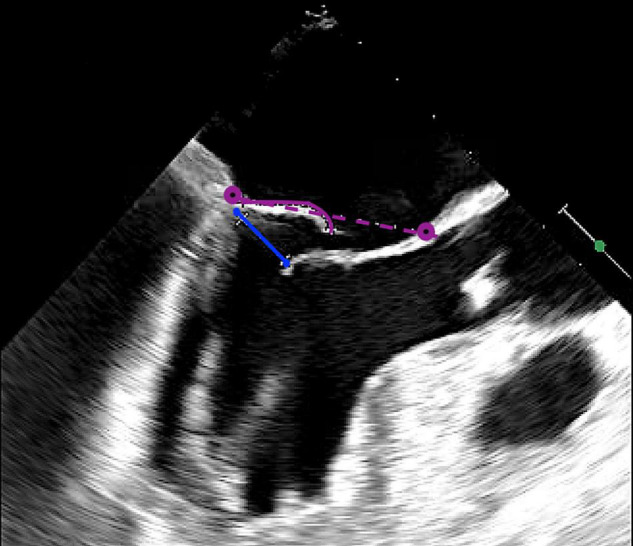
Intraoperative long-axis TEE view showing part of the left atrium, the left ventricle and the left ventricular outflow tract, before repair with the HARPOON device. The measurement of the tissue/gap-ratio is demonstrated. Tissue-length, 1.98 cm; gap-length, 1.14 cm. Tissue/gap-ratio = 1.98/1.14 = 1.74. The anteroposterior diameter is 3.76 cm.

We report the selection criteria based on the current practice as applied in our center (Table 2). Patients are considered eligible for a Harpoon BHMVR procedure in case of severe DMR due to mid-segment pMVL (P2) prolapse, as determined on echocardiography. Although transthoracic echocardiography (TTE) is often used for the primary diagnosis, complementary transesophageal echocardiography (TEE) (e.g., with Philips EPIQ 7) including 3D clips is required to meticulously identify the mechanism of MR and extent of the prolapsing segment. In order to reduce MR without undue leaflet tension after cord implantation, the mitral leaflet coaptation surface needs to be sufficient. Sufficient coaptation is defined by a minimum tissue to gap ratio of 1.5:1, measuring the posterior prolapse segment length and the corresponding anteroposterior distance between the free edge of the anterior leaflet and the base of the prolapsed posterior leaflet segment (Figures 2, 3). Patients that have more extensive pMVL prolapse, with involvement of P1 and/or P3, and/or additional anterior leaflet prolapse are thus far not considered eligible for the procedure. Other major exclusion criteria are active endocarditis, left ventricular or left atrial appendage thrombus, severe mitral annular and/or leaflet calcification, mitral stenosis, functional MV disease, previous MV replacement surgery and a fragile or thinning apex. Patients with significant aortic or tricuspid stenosis or regurgitation requiring concomitant cardiac surgery are also not seen as optimal candidates for BHMVR with the Harpoon system.

**TABLE 2:** Screening criteria for HARPOON BHMVR.

Inclusion criteria
– ≥18 years
– Severe MR on TTE
– Mitral leaflet coaptation surface is sufficient to reduce MR without undue leaflet tension (approximate leaflet to gap ratio of at least 1.5:1)
– Degenerative mitral valve disease with mid-segment P2 prolapse

**Exclusion criteria**

– Subject is of age where further growth is expected
– Active endocarditis
– Left ventricular or left atrial appendage thrombus
– Severe mitral annular and/or leaflet calcification
– Cannot tolerate procedural anticoagulation or post-procedure antiplatelet regimen
– Mitral stenosis
– Functional MV disease
– Previous MV replacement surgery
– Fragile or thinning apex
– Contra-indications to TEE
– Subject is pregnant or lactating

*BHMVR, Beating heart mitral valve repair; MR, mitral regurgitation; MV, mitral valve; TEE, transesophageal echocardiography; TTE, transthoracic echocardiography.*

Of note, patients are always prepared conform standard preoperative cardiac surgery work-up, including coronary angiography and laboratory work-up, according to the guidelines.

### Procedural Technique

We will describe the procedure step-by-step, based on the techniques as described by Gammie et al. (36, 37).

#### Patient Set-Up

The patient is prepared for a BHMVR with the Harpoon TSD-5 (Edwards Lifesciences, Irvine, CA) according to the hospital’s standard procedures. The procedure is performed under general anesthesia with a single-lumen endotracheal tube. A cell salvage system, standby cardiopulmonary bypass in case of conversion to port-access surgery, external defibrillator pads and meticulous control of activated clotting time (ACT) are added to enhance patient safety. A monitor that displays the procedural echocardiographic images is placed directly in front of the surgeon on the right side of the patient.

#### Intraoperative Imaging

Intraoperative TEE (e.g., with Philips EPIQ 7 cardiology ultrasound system) is required throughout the entire procedure to guide the insertion of the introducer and delivery system, the subsequent steering and thereafter the deployment of the delivery system. Every procedure should therefore be conducted in close cooperation of a trained cardiothoracic surgeon and experienced imaging cardiologist.

TEE assessment of the MV should be performed before insertion of the introducer, especially when the previous baseline TEE assessment was performed more than a month preoperatively. This enables identification of any changes in the MV pathology and the mechanism of MR and therefore any possible deviations from standard patient eligibility.

#### Incision

The optimal intercostal space (usually the fourth or fifth left intercostal space) is identified with the use of TTE. A small incision (approximately 5 cm) is made parallel to the rib in order to achieve a small left anterior (antero-lateral) thoracotomy.

#### Intrathoracic Exposition

A soft tissue retractor is recommended for use to avoid iatrogenic trauma due to rib spreading (Alexis Wound-protector, Applied Medical, Santa Margarita, CA, United States). After opening the pericardium, four pericardial stay-sutures need to be placed in the most medial and lateral parts of the thoracotomy, respectively, to ensure optimal exposure of the LV.

#### Transesophageal Echocardiography-Guided Introduction of Introducer

**FIGURE 4:**
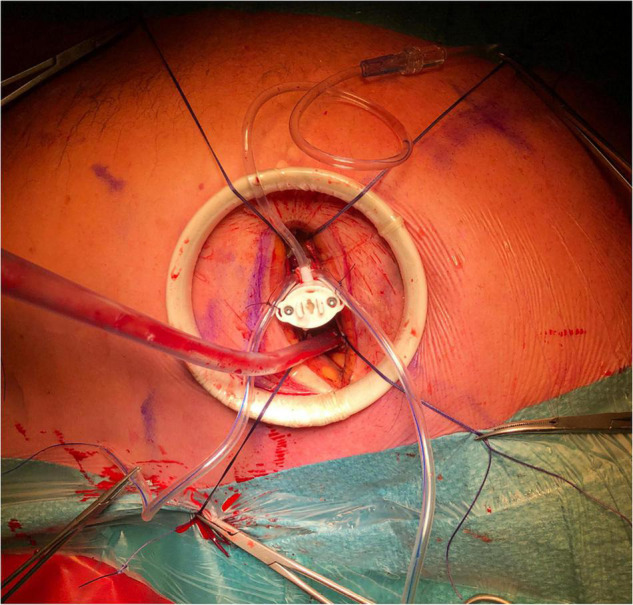
The set-up for Harpoon Beating Heart Mitral Valve Repair through a left anterolateral thoracotomy, as performed in the Sint Antonius Hospital Nieuwegein. The left shoulder and elbow are slightly flexed dorsally. A soft tissue retractor is used without additional rib spreading. Four pericardial stay sutures are placed. The hemostatic introducer is inserted and secured with two concentric purse-string sutures.

The entry-location for the insertion of the introducer must be identified on the epicardium (Figure 4). This should be approximately 3 cm basal from the true apex and 1 cm lateral to the left anterior descending (LAD) coronary artery (i.e., between the LAD and the diagonal branches). The chosen entry-location can be checked with a finger-indentation test on the ventricular wall on TEE to ensure that the introducer will be inserted just apical to the base of the anterolateral papillary muscle. The ME mitral commissural view best visualizes the papillary muscles with the corresponding native chords (the anterolateral papillary muscle is located in the right side of the image).

After determining the entry-location, two concentric pledgeted Gore-Tex purse-string sutures should be placed in the myocardium. Note of caution: the use of Prolene 3-0 sutures is inadvisable, because of the risk of possible tearing of the crossing Gore-Tex sutures. At this moment, intravenous heparin should be administered to achieve an ACT above 350 s.

The hemostatic introducer (with 14-Fr external and 12-Fr internal diameter) is now ready to be inserted using a 0.035” guidewire. Insertion of the introducer should be visualized simultaneously in both the intercommissural and LAX views. On the mitral commissural view, the medial to lateral view of the mitral valve (P3-A3-A2-A1-P1) is seen from left to right. On the long-axis view, the posterior to anterior view (P2-A2) is visualized from left to right.

#### Transesophageal Echocardiography-Guided Introduction of Delivery System

The preloaded delivery system is inserted through the introducer. The delivery system is meant for single-use and has a 9-Fr external diameter (3 mm). During insertion (and withdrawal) of the delivery system, hemostasis is maintained with the internal valve of the introducer (36, 37).

At the moment of insertion, it is of great importance that the delivery system can be immediately located on TEE 2D images in bicommissural and long-axis views simultaneously at all times (e.g., until the introducer is withdrawn after the final knot).

In order to avoid transversing the native chordae tendinae of the aMVL, the delivery system should be first steered in the direction of the posterior leaflet after insertion.

#### Transesophageal Echocardiography-Guided Chord Implantation

Guided by the instructions of the imaging cardiologist, the delivery system is steered and advanced toward the targeted location of the underside of the prolapsed segment of the pMVL. The ePTFE knots should be targeted close to the free edge of the pMVL and should be spaced 3–5 mm apart across the free edge of the prolapsed segment. The knots need to be placed sequentially from lateral to medial.

**FIGURE 5:**
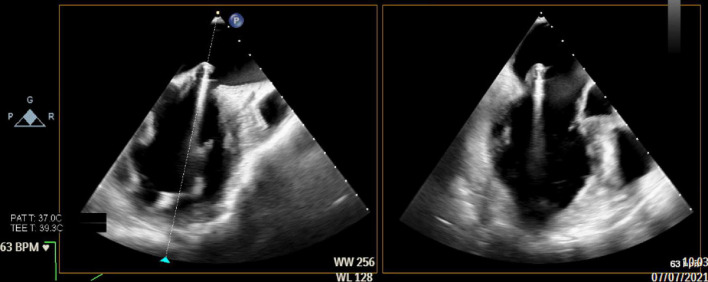
The end effector is used to stabilize the HARPOON device on the underside of the pMVL at the targeted implantation site. pMVL, posterior mitral valve leaflet.

When the delivery system has reached the targeted location, the atraumatic end effector on the distal tip of the delivery system should be used to stabilize the leaflet throughout the cardiac cycle on the underside of the pMVL at the targeted implantation site (Figure 5) (37). A Valsalva test should simultaneously be performed by the anesthesiologist. When the location is satisfactory according to the surgeon and imaging cardiologist, the delivery system can be deployed. When the delivery system is deployed, a 21-gauge needle with a prewrapped ePTFE double-helical knot (50 winds) penetrates the pMVL. After deployment, the needle is automatically withdrawn and the ePTFE coil is tightened, forming a double-helix knot on the atrial leaflet surface (36, 37).

**FIGURE 6:**
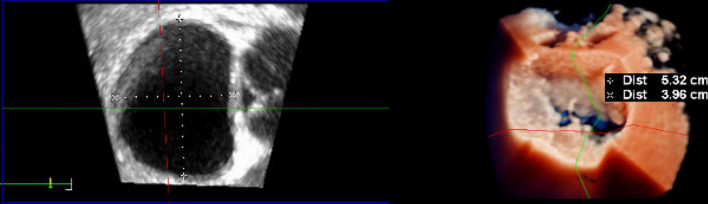
Intraoperative three-dimensional TrueVue photo-realistic rendering of the mitral valve before repair with the HARPOON System. A surgical view of the atrial side of the mitral valve is shown on the right. The anteroposterior diameter of the mitral valve is 3.96 cm.

After each deployment of the device, knot position should be checked with 3D (“surgical”) TEE view (Figure 6), visualizing the mitral valve as seen from the left atrium. The upper leaflet in this view is the anterior leaflet, the lower leaflet in this view is the posterior leaflet. The scallops are seen from left-to-right as follows: A1/P1-A2/P2-A3/P3.

The delivery system is withdrawn from the introducer after each deployment, leaving the two corresponding ePTFE strands exteriorized through the introducer lumen. In order to recognize the ePTFE strands from the following strands to the corresponding number of deployment, the end of the strands can be tied with the corresponding number of deployment (for recognition later in the procedure).

A new delivery system needs to be inserted for each desired chord implantation. Usually three to five knots are implanted, with a minimum amount of three knots. After the final cord implantation has been performed, the introducer can be withdrawn from the LV. To imitate the final result on TEE, the ePTFE strands should be kept under tension.

#### Transesophageal Echocardiography-Guided Tensioning and Correction of Mitral Regurgitation

The purse-string sutures should then be tied and hemostasis needs to be achieved. Hereafter the ePTFE suture pairs are passed separately through a stiff pledget and then through a tourniquet. Under TEE guidance, all ePTFE sutures are tightened simultaneously, titrating the length of the implanted ePTFE chords based on optimal coaptation and minimal MR. Care should be taken that all the implanted chords are evenly tensioned, are not traversing any native chords of the aMVL or pMVL and most importantly are not too over-tensioned. Adequate coaptation (5 mm or more) should be created and residual MR > mild should not be accepted. When the optimal length of the ePTFE chords is achieved, the chords can be fixated with a clamp protected by rubber shuts. Each pair should then be tied over the pledget and cut distally.

#### Closure

The pericardium is left open completely. Routine closure is achieved in layers and a single small caliber left pleural drain (21 Fr) is placed.

Aspirin should be administered postoperatively and daily thereafter until 3 months postoperatively.

### Results of Previous Studies

So far, the results of two studies were published that have investigated the outcome of BHMVR with the HARPOON device in-human, including the Early Feasibility Study and the TRACER CE Mark Trial (36, 38). At the moment, the multi-center ASCEND trial and REPLICATE trial are ongoing and therefore results have not yet been published.

The Early Feasibility study was performed in two different centers in 13 patients from February 2015 until February 2016 (37, 38). In the TRACER Trial, 52 patients were prospectively included in six different centers across Europe (36, 37). Mean age of the patients of 61 ± 12 years and were predominantly male (76%). Mean BMI was 25–26 ± 4 kg/m^2^ and a EuroSCORE II of 1.2 ± 1.1% (37). The degree of MR was evaluated according to the criteria as specified by the American Society of Echocardiography: none, trace, mild, moderate, severe (0–4 +). At baseline, 93% of patients had severe MR and 7% of patients had moderate MR (37).

The latest report of Gammie et al. (37) on the 1-year follow-up data of both trials revealed a procedural success rate of 91% (*n* = 59/65) across both trials, which was defined by successful implantation of ≥ 1 ePTFE chords on the pMVL and demonstration of MR reduction from severe to moderate or less directly postoperatively and after 30 days. In total, two patients were converted to median sternotomy early in the study (study patient numbers 2 and 3) because of inadequate imaging equipment resulting in suboptimal artificial chordal placement. Another procedure was terminated directly after placement of the introducer due to high intra-cavitary LV pressures because of left ventricular outflow tract obstruction.

In treated patients, an average of 4.1 ± 1.0 pairs of ePTFE chords were implanted in the pMVL. Reported mean procedural time was 126 ± 36 min and the introducer remained in the left ventricle for 42 ± 18 min. Patients were discharged from the hospital after 6 ± 2.1 days.

Importantly, MR was reduced to ≤ mild in 95% of patients at discharge, 85% at 30 days, 81% at 6 months and 75% at 1 year postoperatively, excluding reoperated patients. In total, 8 of 62 patients (13%) were reoperated during 1-year follow-up. Reasons of reoperation were postoperative endocarditis (*n* = 1), recurrent MR due to ruptured ePTFE chords on postoperative day 211/253/352 (*n* = 3) and recurrent prolapse on day 279 due to invagination of the ventricular free wall below the pledget at the entrance site (*n* = 1) (37). One patient required conventional cardiac surgical reoperation for recurrent symptomatic severe MR on postoperative day 72 and one of the ePTFE cord pairs was found to have become untied from the epicardial pledget (38). Causes of recurrent MR were multifactorial in two cases, including unrecognized aMVL prolapse, native anterior chordal rupture and an untied ePTFE knot at the apex (37).

Favorable LV remodeling occurred across groups with a significant decrease of left ventricular end-diastolic dimension (LVEDD) by 11% (from 53 ± 5 mm to 47 ± 6) and a significant decrease of left ventricular end-diastolic volume (LVEDV) by 22% (from 153 ± 41 ml to 119 ± 28) at 1-year follow-up (37). The mean left ventricular end-systolic volume (LVESV) was reduced from 52 ± 20 to 45 ± 14 ml at 6 months follow-up (*p* < 0.001) in the TRACER trial, but was not significantly reduced in the early feasibility study (43 ± 16–42 ± 20) (36, 38). A significant reduction in MV annular AP diameter was reported from 34 ± 5 to 31 ± 5 mm at 1-year follow-up (*p* < 0.001) in pooled data of both trials (37), and a mitral annular area reduction from was found from 10.0 ± 2.7 to 6.9 ± 2.0 cm^2^ at 6-month follow-up in the TRACER trial (36). MV annular gradient remained low in both trials, with a mean of 1.4 ± 0.7 mmHg at 1-year follow-up (37).

Additionally, there was positive reverse remodeling of the left atrium with significant decreases in LA volumes both in the early feasibility study at 30 days (reduction from 117 ± 73 to 85 ± 68 ml) and at a longer follow-up period 6 months postoperatively in the TRACER trial [from 106 ± 36 to 69 ± 24 ml at 6 months (*p* < 0.001)] (36).

Although chordal replacement is regularly recommended in combination with a ring annuloplasty in case of DMR, this technique does not allow for additional annuloplasty. However, by strict selection of patients with a tissue-to-gap ratio of at least 1.5:1 (thus in the absence of severe annular dilation), sufficient mitral leaflet coaptation surface can be created after chordal placement in the P2 segment. Additionally, the AP diameter is also slightly reduced by this technique. Despite the fact that the durability of repair still has to be proven with future studies, the impact of this procedure is relatively low compared to on-pump mitral repair, especially in elderly patients.

## Clinical Perspective and Implications for the Future

Minimally invasive MV repair of mid-segment pMVL prolapse by implantation of ePTFE artificial chords has proven to be a safe and equally durable repair technique as the classic (Carpentier-based) leaflet resection strategies by full median sternotomy, with the added value of less surgical trauma, improved cosmesis and faster postoperative recovery. Novel “least invasive” transapical transcatheter Beating Heart Mitral Repair technologies have the potential to provide an equally effective anatomical repair in selected patients with the added advantage of a real-time TEE-guided fine-tuning of the result on the beating heart. Whether or not the absence of an annuloplasty ring in selected patients with sufficient TGR will impact on mid to long term durability remains to be seen. Moreover, the reduced surgical trauma in this least invasive MVr technique should be weighted against the durability of the repair, which can be of value especially in elderly higher risk patients. Failure of the transapical BHMVR does not exclude the option of either redo BHMVR or port-access MV repair or replacement. In the end, it is all about weighing risks and benefits. Early results are promising, but long-term larger-scale studies—which are ongoing—are needed and eagerly awaited.

## Author Contributions

All authors listed have made a substantial, direct, and intellectual contribution to the work, and approved it for publication.

## Conflict of Interest

The authors declare that the research was conducted in the absence of any commercial or financial relationships that could be construed as a potential conflict of interest.

## Publisher’s Note

All claims expressed in this article are solely those of the authors and do not necessarily represent those of their affiliated organizations, or those of the publisher, the editors and the reviewers. Any product that may be evaluated in this article, or claim that may be made by its manufacturer, is not guaranteed or endorsed by the publisher.

## Abbreviations

ACT: Activated clotting time
aMVL: Anterior mitral valve leaflet
AP2CH: Apical two-chamber
AP3CH: Apical three-chamber
AP4CH: Apical four-chamber
AP5CH: Apical five-chamber
AVJ: Atrioventricular junction
BHMVR: Beating Heart Mitral Valve Repair
CPB: cardiopulmonary bypass
CT: Computed tomography
DMR: Degenerative mitral regurgitation
ePTFE: Expanded polytetrafluoroethylene
Fr: French
ICS: Intercostal spacel
LA: Left atrium
LAD: Left anterior descending
LAI: Leaflet-to-annulus index
LAX: Long-axis
LV: Left ventricle
LVEDD: Left ventricular end-diastolic dimension
LVEDV: Left ventricular end-diastolic volume
LVESV: Left ventricular end-systolic volume
MA: Mitral annulus
MAC: Mitral annular calcification
MAD: Mitral annular disjunction
MIMVr: Minimally invasive MV repair
MIMVS: Minimally invasive mitral valve surgery
MR: Mitral regurgitation
MV: Mitral valve
PISA: Proximal Isovelocity Surface Area
PLAX: Parasternal long-axis
pMVL: Posterior mitral leaflet
PSAX: Parasternal short-axis
SAM: Systolic anterior motion
TEE: Transesophageal echocardiography
2D: Two dimensional
3D: Three dimensional.
